# Comparison of the efficacy of the anterolateral thigh flap for perforator localization in the repair of head and neck soft tissue defects patients: A protocol study for a randomized controlled trial

**DOI:** 10.1097/MD.0000000000023080

**Published:** 2020-12-11

**Authors:** Jiangfeng Sheng, Chao Li, Ping Tang, Jun Hu, Liying Ma, Gang Qin

**Affiliations:** a Department of Otolaryngology Head and Neck Surgery, Affiliated Hospital of Southwest Medical University; b Department of Head and Neck Surgery, Sichuan Cancer Hospital; c Department of Thyroid, Head, Nneck and Maxillofacial Surgery, The Third People's Hospital of Mianyang City.

**Keywords:** anterolateral thigh flap, CT angiogram, head and neck soft tissue defects, MIMICS, perforator localization

## Abstract

**Background:**

Head and neck tissue defects cause great physical and psychological damage to patients. Therefore, accurate positioning of perforating vessels before operation is of great significance for improving the success rate of flap preparation and avoiding unnecessary incision injury.

**Methods/Design:**

A total of 60 patients with laryngeal cancer in otolaryngology, Department of Otolaryngology Head and Neck Surgery, Affiliated Hospital of Southwest Medical University and the Third People's Hospital of Mianyang city from October 2020 to October 2021 will be selected and randomly divided into CT angiogram (CTA) group (n=20), mimics group (n = 20) and CTA + mimics group (n = 20) according to the numerical table. Patients in the CTA group will receive CTA examination of lower extremities. Patients in mimics group will receive digital technology in the positioning of perforator. Patients in CTA + mimics group will receive CTA + digital technology. All the patients will receive the flap cutting and the flap making; the doctor will determine the perforation branch of the flap with 3-D visual positioning, measure the preoperative indicators intraoperatively and complete the wound repair. Finally, the survival rate, sensitivity, specificity and accuracy of the flap will be measured.

**Discussion:**

The anterolateral thigh flap has been widely used to repair various tissue defects and has obtained good clinical results. The extensive clinical application mainly focuses on 2 aspects, namely the study of vascular anatomy of lateral flap and the exploration of preoperative flap design technology. Perforator is the direct blood supply source of anterolateral thigh flap, so it is particularly important to study the anatomy of perforator. Therefore, this study will reveal CTA combined with digital technology in the vascular anatomy of the anterior external femoral flap and the design of the flap before and during surgery, so as to provide help for the repair of tissue defects.

**Trial registration:**

It has been registered at http://www.chictr.org.cn/listbycreater.aspx (Identifier: ChiCTR2000038951), Registered on October 10th, 2020.

## 1 Introduction

Head and neck tissue defects are often caused by trauma or infection caused by tumor resection.^[1]^ Tissue defects in the head, neck, maxillofacial and oral cavity lead to facial damage, and affect the patient's breathing, chewing, swallowing, speech and other physiological functions, thus affecting the patients quality of life.^[2]^ Therefore, the reconstruction of head, neck and maxillofacial tissue defects and the ideal reconstruction of morphology and function are urgent problems for surgeons. At present, the proximal pedicle flap repair is a common way to repair head and neck soft tissue defect. However, for patients with large defects and near tumor metastasis, the operation of adjacent pedicled tissue flap is limited.^[3]^ With the rapid development of microsurgical techniques, vascular anastomosis free tissue flap repair has been widely used, and the success rate of vascular anastomosis has gradually increased.^[3,4]^ The anterolateral thigh flap (ALTF) has been most widely used for reconstruction of head and neck defects.^[5]^

Interactive control system software-Materialises interactive medical image control system (MIMICS) has been broadly applied in medical researchers,^[6,7]^ some scholars tried to apply 3-D to perforator localization and the design of the flap, flap perforators through the analysis of the 3-D visualization of blood vessels of the origin, contorts, within the muscle skin distribution relationships, and rebuild the precise 3-D visualization flap model.^[8]^ CT angiogram (CTA) technology combined with 3-D software has gradually been used to study vascular anatomy of flaps and preoperative flap design, its feasibility and some advantages have been reported.^[9]^ However, such studies are not yet mature, and there are few clinical studies on the preparation of anterolateral thigh flaps, so the feasibility remains to be further explored and improved.

## 2 Methods/design

### 2.1 Research object

We aim to compare the efficacy including survival rate, sensitivity, specificity and accuracy of in patients divided into CTA group, mimics group and CTA + mimics group in the repair of head and neck soft tissue defects with anterolateral thigh flap.

### 2.2 Study method

1. The patients will complete routine preoperative examination to exclude serious systemic diseases, and received enhanced valvular CT scan, so that the doctor could determine the lesion scope and vascular condition in the recipient area;
2. The patients will be placed in the supine position, the middle and upper 1/3 of both lower extremities will be radiographically marked, and both lower extremities will be kept naturally straight. Iohexol injection will be injected intravenously from the middle of the elbow. A 256-slice spiral CT (Philips, USA) will be used for continuous CTA scanning from bilateral anterior superior iliac spine to bilateral lower edge of patella. CT scanning parameters will be set as 0.625 mm thickness, 120-140 kV, and 525 mA.
3. CTA data preprocessing, search for puncture and define reconstruction area: obtain CTA data in DICOM format and import CTA data into 3-D15.0 software (Materialize, Belgium). After reading the data, 3-D15.0 software automatically creates a 3-D project file and uses the convert function to complete the data conversion and generate the original CTA image. Based on the original cross section, the software automatically reconstructs the continuous sectional images of sagittal plane and coronal plane to form the observation screen of 3 dimensions. The original CTA images will be scanned and observed in three orthogonal sectional views to determine the appropriate location of the perforation of the anterolateral femoral flap. Then, the 3-D reconstruction area will be restricted to 1 side of the supply area with suitable cutting struts with Crop Mask, thus reducing the workload of data segmentation in later period.
4. 3-D is used to reconstruct bones, blood vessels and skin: a section line is created by using the section line option in the axial window, and the gray value distribution along the line can be obtained. To better observe the transition of soft tissue, cortical bone and cancellous bone, click the option of Start Thresholding and select a certain amount of pixel gray value to segment the bone from anterior superior iliac spine to ipilateral patella. The soft tissue and some discrete voxels are then separated from the bone by means of regional growth. Select the region growth tool from the segmentation toolbar and click on the bone section to add all associated voxels to the new mask. The hip, femur, and patella will be extracted from the shape and outline of the bone at each axis view level using a multi-layer editing tool. The newly added mask will be reconstructed in 3-D to extract the individual bone model. Adjust appropriate thresholds to create a new mask, use a multi-layer editing tool on the axis view to erase irrelevant grayscale values, and retain and extract major blood vessels and perforations. The erased new mask will be reconstructed in 3-D, and the reconstructed plane mask will be duplicated. Use the Edit Mask in 3-D to edit the reconstructed 3-D model. Remove the irrelevant soft tissue with the cover lock tool. Click the erase tool to remove the selected pixels from the Mask. After the 3-D editing function is completed, close the toolbar and carry out 3-D reconstruction on the edited corresponding plane mask, so as to obtain clear blood vessels and perforating branches. Reconstruct the thigh skin, create a new mask, set the segmentation threshold, erase the gray value of irrelevant structure on the axis view, carry out 3-D reconstruction, and use the wrap function to make the reconstructed skin continuously smooth.
5. 3-D visualization of the model: give bone and blood vessel an opaque and skin transparency High, which allows observation and determination of bone and blood vessel through the skin. To enhance the visual effect, the target perforations and pedicles can be stained to a specific color. Based on the size and shape of the wound in the receiving area and the cutting function in the simulation option, the scope, depth and path of the flap will be simulated along the skin surface to generate a 3-D visual model of the personalized free anterolateral thigh flap.
6. Simulated skin flap removal and operation: 3-D will be used to edit and process the reconstructed 3-D model before surgery. After other parts will be hidden, the designed anterolateral thigh flap will be “removed” from the 3-D model together with the vascular pedicle. The simulated data will be transferred to the intraoperative body surface of the patients, and the flap will be surgically removed to verify the accuracy of perforator location, and the anterolateral thigh flap will be transplanted and repaired for head and neck soft tissue defects.
7. Routine nursing care after free flap transplantation will be given to promote wound healing. Preoperative and intraoperative measurement data will be processed and analyzed with SPSS20.0 software.

### 2.3 Participants

Inclusion criteria include the following:

1. A total of 60 patients with laryngeal cancer in otolaryngology, Department of Otolaryngology head and Neck Surgery, Affiliated Hospital of Southwest Medical University and the Third People's Hospital of Mianyang city from October 2020 to October 2021 will be selected, aged from18 to 65 years old, regardless of gender;
2. Head and neck with bone and soft tissue exposure, or tumor resection after skin grafting cannot repair the wound surface;
3. Can fully understand the surgical risks and sign informed consent and ethical consent;

Exclusion criteria include the following:

1. Children, adolescents less than 18 years of age, pregnant women and elderly patients with apparent physical decline;
2. Patients with positive iodine allergy test and abnormal renal function;
3. Patients with severe temperament diseases who cannot tolerate surgery;
4. patients with mental illness who cannot independently sign the informed consent for the operation;

### 2.4 Interventions

Patients will be randomly divided into CTA group (n = 20), mimics group (n = 20) and CTA + mimics group (n = 20) according to the numerical table. Patients in the CTA group will receive CTA examination of lower extremities. Patients in mimics group will receive digital technology in the positioning of perforator. Patients in CTA + mimics group will receive CTA + digital technology. All the patients will receive the flap cutting and the flap making; the doctor will determine the perforation branch of the flap with 3-D visual positioning, measure the preoperative indicators intraoperatively and complete the wound repair. Finally, the survival rate, sensitivity, specificity and accuracy of the flap will be measured.

### 2.5 Measurement of outcomes

Vascular anatomy and preoperative design results of the anterolateral thigh flap will be recorded, and intraoperative and preoperative comparisons will be analyzed to explore the clinical efficacy of postoperative patients. After the completion of image digitization, 2 physicians who have been working on the anterolateral thigh flap for more than 4 years read the images in a double-blind way to evaluate whether the location, shape, and scope of the lesion of the patient has invasion or metastasis. When opinions will be different, they read the images together and agreed. Consistency analysis by CTA joint digital technology, digital technology and CTA in the defects of head and neck cancer patients in the orientation of value, when the CTA inspection found perforators preoperative and intraoperative confirmed existence is judged to be (+), when the digital technology can be worn for successful reconstruction is judged to be (+), either in the joint inspection tests to determine the (+), finally to the 3 ways to wear the positioning performance comparison.

### 2.6 Statistical analysis

Statistical analyses will be implemented by SPSS 17.0 and Microsoft Excel 2007 software. Data will be represented as mean ± standard deviation (SD). A *t*-test will be performed to compare the changes in measures within groups. Statistical significance will be considered at *P* < .05.

## 3 Discussion

The anterolateral thigh flap has been widely used to repair various tissue defects and has obtained good clinical results. The extensive clinical application mainly focuses on 2 aspects, namely the study of vascular anatomy of lateral flap and the exploration of preoperative flap design technology. Perforator is the direct blood supply source of anterolateral thigh flap, so it is particularly important to study the anatomy of perforator. Therefore, this study will reveal CTA combined with digital technology in the vascular anatomy of the anterior external femoral flap and the design of the flap before and during surgery, so as to provide help for the repair of tissue defects.

## Author contributions

Jiangfeng Sheng conceived the idea for this study; Chao Li and Ping Tang provided statistical plan; Gang Qin drafted the protocol. Jun Hu and Liying Ma reviewed the protocol and provided critical feedback. All authors approved the article in its final form.

**Conceptualization:** Jiangfeng Sheng, Chao Li.

**Data curation:** Liying Ma.

**Formal analysis:** Liying Ma.

**Funding acquisition:** Jiangfeng Sheng.

**Methodology:** Ping Tang.

**Project administration:** Jiangfeng Sheng.

**Software:** Chao Li, Ping Tang.

**Supervision:** Jun Hu.

**Validation:** Jun Hu.

**Writing – original draft:** Jiangfeng Sheng, Gang Qin.

**Writing – review & editing:** Gang Qin.
